# Factors correlating to the propensity of general practitioners to substitute borderline vitamin B12 deficiency

**DOI:** 10.1080/02813432.2018.1487522

**Published:** 2018-06-22

**Authors:** Grace Cham, Nichola Davis, Edward Strivens, Aileen Traves, Grant Manypeney, Ronny Gunnarsson

**Affiliations:** a Cairns Clinical School, College of Medicine and Dentistry, James Cook University, Australia;; b Older Persons and Subacute Services, Cairns and Hinterland Hospital and Health Service, Cairns, Queensland, Australia;; c Mareeba Medical Clinic, Queensland, Australia;; d Research and Development Unit, Primary Health Care and Dental Care, Southern Älvsborg County, Närhalsan, Västra Götaland Region, Sweden;; e Department of Public Health and Community Medicine, Institute of Medicine, The Sahlgrenska Academy, University of Gothenburg, Sweden

**Keywords:** Vitamin B12 deficiency, dietary supplements, general practice, primary health care

## Abstract

**Objective:** This study aims to identify factors which correlate to the propensity of general practitioners (GPs) to prescribe supplementation for borderline vitamin B12 deficiency.

**Design:** Cross-sectional surveys were distributed in person.

**Setting:** Conferences held in Cairns, Palm Cove Beach, Mt Isa; educational meetings in Atherton; and meetings with individual general practices within the Cairns and Hinterland region. All located in Queensland, Australia.

**Subjects:** 128 practicing GP specialists and registrars (practitioners in training).

**Main outcome measures:** Responses to the Likert scale statements with its five options scaling from ‘strongly disagree’ to ‘strongly agree’ were recoded to have binary outcomes for analysis.

**Results:** A survey response rate of 89% was achieved. Participants who felt patient demands influence the management of borderline vitamin B12 deficiency were more likely to prescribe supplementation (OR 2.4, *p* = 0.037). Participants who perceived an overuse of vitamin B12 were less likely to prescribe B12 (OR 0.39, *p* = 0.019). Participants who often saw patients with vitamin B12 deficiency were less likely to request for the complementary biomarkers plasma methylmalonic acid or total homocysteine (OR 0.41, *p* = 0.045).

**Conclusions:** The identified disparity to prescribe vitamin B12 for borderline deficiency may be described as an attempt in the GP collective to seek a balance between being the patient’s or the society’s doctor. We propose that relevant authorities try to reduce this disparity by describing a management strategy for borderline vitamin B12 deficiency.

**Key points**
- General practitioners hold different thresholds for commencing supplementation in cases of borderline vitamin B12 deficiency.
- Participants from Australia were asked to fill out a cross-sectional survey to explore factors which correlate with the propensity to prescribe in clinical practice.
- Our study identified that patient demands and a practitioner’s perception of whether there is an overuse of vitamin B12 in the community influenced the propensity to treat for deficiency.
- The results give insight into reasons for initiating supplementation, and will help inform general practitioners on their current management.

## Background

Vitamin B12 deficiency is a public health issue. Although the true prevalence of vitamin B12 deficiency within most populations remains unknown, figures for low serum vitamin B12 are reported up to 23% within the elderly population [1–3]. Other risk groups commonly affected include vegans and vegetarians, individuals of South-Asian heritage, patients with pernicious anaemia or a malabsorption syndrome, those who have undergone gastric surgery and users of metformin, psychotropic drugs or gastric acid-blocking agents [4–10].

Vitamin B12 deficiency is defined by characteristic clinical features which respond to supplementation. As the condition has an insidious onset which can manifest with an array of non-specific haematologic and/or neuropsychiatric signs and symptoms, vitamin B12 deficiency can be a difficult diagnosis. Very low levels of serum B12 (such as below 100 pmol/L) is considered to represent deficiency by most practitioners. Markedly elevated values of serum B12 (such as above 350 pmol/L) are taken to indicate the absence of deficiency, and symptoms are likely attributed to other conditions. There is however no internationally accepted consensus on the upper and lower limits for serum vitamin B12 levels [11].

The value of newer biomarkers, such as plasma methylmalonic acid and total homocysteine, over the total serum B12 assay in diagnosis is contentious. Evidence in the form of randomized controlled trials is lacking, and few international guidelines on the management of vitamin B12 deficiency have been published [12–14]. Guidelines released by The British Committee for Standards in Haematology state that serum B12 should be followed up by second-line methylmalonic acid to clarify uncertainties [14]. However, the latter test is not readily available in Australia [15].

Thus, the diagnosis and management of borderline vitamin B12 deficiency within general practice is shrouded by uncertainty and variable between practitioners. Requests for B12 testing have grown rapidly. Between 2003-2004 and 2013-2014, claims for tests in Australia measuring serum B12 or red cell folate (and serum folate if required) increased by 119% [16]. The most common reasons for requesting tests were macrocytic anaemia, neurological symptoms and screening [17–19].

In contrast, there is no existing literature on the reasons for initiating vitamin B12 supplementation. An earlier qualitative study conducted by the authors revealed five sub-themes which summarised the opinions and attitudes of GPs on borderline vitamin B12 deficiency [20]. Overall GPs emphasised on the lack of quality post-graduate teaching, pressures to prescribe from patients and differing views between colleagues on how deficiency should be managed. Further research on these sub-themes may elicit interesting findings on why supplementation is initiated. This will help to inform GPs on their management and may lead to better patient health outcomes.

This study aims to identify factors which correlate to the propensity of GPs to prescribe supplementation, either as intramuscular injections or in oral form, for borderline vitamin B12 deficiency. We define borderline deficiency as a situation where the GP is uncertain if the patient has a clinically relevant B12 deficiency that should be substituted. We hypothesise that GPs who are more receptive to patient demands correlate with an increased propensity to prescribe B12 supplementation.

## Methods

The study was approved by the Human Research Ethics Committee at James Cook University under the ethics approval number H6476.

### Survey construction

The survey was composed of four parts: demographic information, agreement with constructed statements, approach to a case of borderline B12 deficiency, and when education on deficiency was last received. Participants were required to indicate their age, gender, year of graduation, and level of GP training, but otherwise remained anonymous.

Survey statements and a Likert scale asserting the level of agreement were constructed for the purpose of this study. Statements were based on sub-themes identified in an earlier qualitative study conducted by the authors [20]. The 5-point Likert response scale was employed for the statements and case study, where 1 = strongly disagree, 2 = disagree, 3 = neutral, 4 = agree, and 5 = strongly agree. ‘Neutral’ was retained as an option for participants to select if they felt ambivalent towards a statement, as would be expected for this topic.

A hypothetical case was formulated from the experiences described by participants in an earlier qualitative study: “An 80 year old lady presents with a one year history of fatigue as her sole symptom and a serum B12 level of 195 pmol/L. Physical examination and other pathology results are within normal limits. She takes a short daily walk and has a decent diet.” The practitioner was asked if they would request complementary biomarkers, initiate vitamin B12 supplementation, provide further dietary recommendations or retest in 3-12 months.

With regards to when education was last received, a scale was employed with the options of “more than 10 years ago”, “last 10 years”, “last 3 years”, “last 12 months”, and “last 6 months”. The survey was carefully discussed and revised several times by GC and RG before the final version.

### Data collection

The study population consisted of practicing GPs who were in the Cairns and Atherton Tablelands region in Australia between July and August 2016. Participants were recruited in one of three settings: conferences held in Cairns, Palm Cove and Mt Isa (three occasions), GP registrar educational meetings in Atherton (two occasions), and booked lunch time meetings with individual general practices (20 practices). Surveys were individually numbered for tracking distribution, return and to finally estimate the response rate. These were hand-delivered with information sheets on each occasion, but one, by GC. A total of 15 surveys were distributed, and returned, at a conference in Mt Isa by one volunteering participant. Surveys that had been distributed to practice managers at conferences or left at general practices were followed-up by phone after two weeks, and reminded again one week later. Beyond this timeframe, surveys were marked as unreturned.

### Data analysis

Responses were exported to IBM SPSS Statistics v24 for Windows for statistical data analysis. All variables were recoded to have binary outcomes. For the Likert scale statements, responses marked as ‘strongly disagree, ‘disagree’ or ‘neutral’ were coded as 0, and the responses ‘agree’ and ‘strongly agree’ were coded as 1. For the other scale, education received further back in time than three years was coded as 0, and those within the last three years as a 1. By univariate binary logistic regression, prescription of B12 supplements was analysed as a dependent variable against each other statement. Variables with a level of significance equal or below 0.2 were then taken to multivariate backward stepwise logistic regression. The same process was repeated with the requesting of complementary biomarkers as the dependent variable. The univariate regression was merely used as a sorting mechanism and not considered as a result.

### Sample size calculation

Male medical practitioners have in other situations been seen as more proactive (for better or worse) than female medical practitioners in prescribing behaviour [21]. The authors wanted to explore if this was also true for the prescribing of vitamin B12 supplementation for the described scenario. We assumed that 30% of female and 60% of male practitioners are high prescribers of B12 and using logistic regression. Level of significance was set to 0.05 and power set to 80%. A required sample size of 88 GPs was calculated by the statistical power analysis program G*Power, version 3.1.9.2, on 31st October, 2014. We aimed to collect more than 90 surveys.

## Results

During the seven weeks of data collection from July to August 2016, a total of 144 surveys were distributed and 128 (89%) were returned. There were 99 (77%) fully completed and 29 (23%) partially completed surveys. The majority of surveys were returned in person (*n* = 109, 85%) and 19 were returned by e-mail (15%).

### Participant demographics

Participants’ ages ranged from 26 to 72 years. Of the group, 46% (*n* = 59) were female, 52% (*n* = 67) were male and two participants did not disclose gender. GP specialists made up 63% of participants (*n* = 80).

### Distribution of responses

The majority (63%, *n* = 80) of participants indicated they commonly see patients with vitamin B12 deficiency (Table 1). There was a wide spread of responses towards the influence of colleagues’ practice, and towards patient demands, with almost equal numbers on either side of neutral in both cases (number in disagreement to number in agreement were 43:50 and 49:41, respectively). However, participants generally inclined towards agreement with their colleagues’ practice, and disagreement in response to patients’ preferences. A large proportion (42%, *n* = 54) of participants were neutral to the statement on the overuse of vitamin B12 in Australia. The vast majority of participants (84%, *n* = 108) agreed with the statement that in cases of known malabsorption, injections were advocated over oral therapy. Complementary biomarkers, defined here as either methylmalonic acid or total homocysteine, were often requested by 16% of participants (*n* = 21).

**Table 1.** Responses to statements on borderline B12 deficiency.

Survey Statements	*n*	Strongly Disagree	Disagree	Neutral	Agree	Strongly Agree
I commonly see patients with vitamin B12 deficiency	128	6	23	19	69	11
By discussion or through patient notes, I observe how colleagues manage cases of vitamin B12 deficiency and imitate them	126	9	34	33	46	4
Patient demands influence my management of borderline vitamin B12 deficiency	128	9	40	38	37	4
There is currently an overuse of vitamin B12 in Australia	127	3	17	54	38	15
I advocate injections over oral therapy in patients who require vitamin B12 treatment and have known malabsorption	128	3	8	9	67	41
I often request for complementary biomarkers (methylmalonic acid, homocysteine) prior to diagnosing and treating for vitamin B12 deficiency	128	20	64	23	15	6

In relation to the hypothetical case of borderline B12 deficiency (Table 2), participants were largely supportive of commencing B12 supplementation (51%, *n* = 60), providing dietary advice (79%, *n* = 97) and re-testing levels on follow-up (89%, *n* = 109). A large proportion (44%, *n* = 54) of participants were disinclined to requesting any additional biomarkers; however 24% (*n* = 29) of participants also felt ambivalent towards the statement. It was noted that the statement to ‘recommend or initiate vitamin B12 supplementation’ had the lowest frequency of responses (*n* = 117).

**Table 2.** Responses to hypothetical case of borderline B12 deficiency.

Survey Statements	*n*	Strongly Disagree	Disagree	Neutral	Agree	Strongly Agree
Request complementary biomarkers	123	16	38	29	35	5
Recommend or initiate vitamin B12 supplementation	117	8	25	24	54	6
Provide further dietary recommendations	123	3	12	11	79	18
Retest in 3 – 12 months	123	3	7	4	88	21

A total of 56 participants (44%) received some form of formal teaching on vitamin B12 deficiency within the last three years. For 28 participants (22%), teaching on the topic was more than ten years ago.

### Propensity to request biomarkers or prescribe B12

By univariate analysis with the initiation of B12 supplementation as the dependent variable, three independent variables were found to have a *p*-value of less than 0.2 (Table 3). Two variables remained statistically significant on multivariate analysis: an agreement that patient demands influence the management of borderline vitamin B12 deficiency (*p* = 0.037), and belief in an overuse of vitamin B12 in Australia (*p* = 0.019).

**Table 3.** Factors correlating to the propensity to prescribe B12 supplementation.

	Univariate (unadjusted) logistic regression			Step-wise multivariate (adjusted) logistic regression (*n* = 114)	
	*n*	*p* value	OR (95% CI)	*p* value	OR (95% CI)
Increasing age of GP (decades)	115	0.88	0.97 (0.70 – 1.4)		
Male Gender	115	0.11	0.55 (0.26 – 1.1)		
GP Trainee	109	0.21	1.7 (0.75 – 3.7)		
Commonly sees B12 deficiency	117	0.24	1.6 (0.73 – 3.3)		
Influenced by colleagues	115	0.45	1.3 (0.63 – 2.8)		
Influenced by patients	117	0.052	2.2 (0.99 – 4.8)	0.037	2.4 (1.1 – 5.7)
Perceives overuse	116	0.029	0.43 (0.20 – 0.92)	0.019	0.39 (0.18 – 0.86)
Advocates injections	117	0.53	1.4 (0.50 – 3.8)		
Requests biomarkers	117	0.69	1.2 (0.45 – 3.4)		
Last B12 education <3 years ago	115	0.75	1.1 (0.54 – 2.4)		

With the requesting of complementary biomarkers as the dependent variable, three different independent variables were identified with a p-value of less than 0.2 (Table 4). Two variables remained on multivariate analysis: participants who commonly see patients with vitamin B12 deficiency (*p* = 0.045), and age of the participants (*p* = 0.070).

**Table 4.** Factors correlating to the propensity to request complementary biomarkers.

	Univariate (unadjusted) logistic regression			Step-wise multivariate (adjusted) logistic regression (*n* = 112)	
	*n*	*p* value	OR (95% CI)	*p* value	OR (95% CI)
Increasing age of GP (decades)	121	0.19	1.3 (0.90 – 1.8)	0.070	1.4 (0.97 – 2.1)
Male Gender	121	0.72	1.1 (0.53 – 2.5)		
GP Trainee	114	0.15	0.51 (0.21 – 1.3)		
Commonly sees B12 deficiency	123	0.11	0.53 (0.25 – 1.2)	0.045	0.41 (0.17 – 0.98)
Influenced by colleagues	121	0.66	1.2 (0.55 – 2.6)		
Influenced by patients	123	0.68	1.2 (0.53 – 2.6)		
Perceives overuse	122	0.25	1.6 (0.73 – 3.3)		
Advocates injections	123	0.53	0.72 (0.26 – 2.0)		
Last B12 education <3 years ago	121	0.57	1.3 (0.58 – 2.7)		

## Discussion

### Summary of main findings

The present study identified factors which correlate to GPs’ propensity to prescribe for borderline vitamin B12 deficiency. Practitioners who were more receptive to patient demands were found to be more likely to prescribe B12 supplementation than their counterparts. Meanwhile, practitioners who perceived there to be an overuse of B12 supplementation in Australia were less likely to prescribe. The use of complementary biomarkers prior to diagnosing or treating for deficiency was limited amongst participants. The present study found that GPs who commonly see patients with vitamin B12 deficiency were less likely to submit requests for complementary biomarkers.

### Being the patient’s or the society’s doctor

Our study findings echo the major theme of ‘Balancing’ described by Hansson et al. in their descriptive, interview-based study conducted in 2007 [22]. In the present study, GPs who are receptive to patient demands for testing or treatment are more likely to initiate B12 supplementation. We interpret this finding as GPs prescribing in the interest of the patient. Conversely, participants who felt there is an overuse of B12 supplementation were, almost to the same degree, less likely to prescribe. These GPs appear to place a greater emphasis on the potential impact of prescription to the wider community.

The continuous struggle of finding a balance between being the personal doctor and the society’s doctor is described by Hansson et al. as a source of inner conflict. Due to the nature of primary health care, GPs are in a unique position where they can develop very strong, continuous doctor-patient relationships. At the same time, there is also a sense of duty that has to be upheld towards society. It is not known whether vitamin B12 testing is cost-effective, as no economic analyses on the subject have been conducted [16]. However, testing on the national scale has rapidly increased in recent years, and repeated testing for previously indeterminate results could lead to financial costs and anxiety to the patient. These considerations need to be weighed with respect to patients’ wishes, and thus GPs have to find a balance between these opposing conditions. In the absence of guidelines with a strong evidence base, GPs need to be aware of how their prescribing propensity may be affected by their acceptance and response to these roles.

### Complementary biomarkers

Study participants were generally disinclined or felt ambivalent towards the use of complementary biomarkers prior to diagnosing and managing borderline vitamin B12 deficiency. The association was statistically significant for GPs who regularly saw patients with vitamin B12 deficiency. We propose two explanations for this finding. Firstly, these GPs see patients with more severe clinical manifestations of deficiency and therefore warrant immediate supplementation, even if serum B12 levels are within normal limits or the indeterminate range [14]. Secondly, GPs who regularly see patients on the spectrum of deficiency are more familiar with the limitations around complementary tests. Although plasma methylmalonic acid is commonly used overseas as a second-line investigation to clarify uncertainties, the test has not been found to be diagnostically accurate and also incurs a cost in Australia, making it less used [14,23].

Furthermore, GPs’ adherence to guidelines varies. In a recent qualitative meta-analysis, GPs’ attitudes and adherence to guidelines were found to be systematically influenced by whether the guideline of interest is prescriptive or proscriptive. Guidelines were not believed to be flexible enough or to accurately reflect the complexity of individual cases [24]. Thus, despite international recommendations, local GPs may continue to doubt the value of complementary tests.

Existing literature suggest increasing age is associated with lower rates of pathology ordering [25]. In contrast, our study found the propensity to request complementary biomarkers appeared to increase with respect to age. Although the *p* value was 0.07, this is potentially interesting. One possible explanation may be that older GPs have spent more years in clinical practice and have experienced more missed cases of borderline B12 deficiency (where patients later became symptomatic) that they now want to identify with the newer complementary tests.

### Study strengths

The present study achieved a response rate of 89%, which is high for GP surveys. This is in contrast to the average response rate of 61% (95% CI 59-63%) for postal surveys [26]. Brodaty et al. outline time as the main barrier to GP participation [27]. As such, to avoid this barrier commonly found with postal or online distribution, a decision was made to hand deliver and collect surveys in-person. Survey statements were designed from the results of a qualitative study previously conducted by the same authors. Data collected from the quantitative part were thus more likely to reveal significant information that may confirm the opinions and attitudes revealed in the qualitative study.

### Study limitations

Due to the indiscriminate recruiting of GPs at conferences, our study is not representative of all GPs who practice in the Cairns and Atherton Tablelands region, Australia. However, this was a deliberate decision made to encourage participation in the study. Although surveys were hand-delivered so questions could be clarified at the scene, there is still a possibility that participants may have experienced some confusion over the wording or meaning of the statements, but chose to not verbalise this confusion.

### Application and future research

To our knowledge, this study is the first to examine factors correlating to GPs’ prescribing propensity of supplementation for borderline vitamin B12 deficiency. As outlined in the preceding qualitative research, GPs are often unaware of how colleagues practice, but at the same time, use colleagues’ documentation as a comparative marker to determine the appropriateness of their management [20]. The present study findings enable GPs to compare and actively reflect on their prescribing behaviour towards vitamin B12 supplementation. Similar research to this study can be conducted with GPs in other regions of Australia or overseas to confirm the present study’s findings.

## Conclusion

Borderline vitamin B12 deficiency is common amongst patients who present to general practice. The ability to recognise, diagnose and treat for deficiency however, is variable between GPs. When faced with borderline vitamin B12 deficiency, GPs were largely agreeable with initiating supplementation, providing dietary advice and re-testing serum vitamin B12 levels on follow-up. The identified disparity to prescribe vitamin B12 for borderline deficiency may be described as an attempt in the GP collective to seek a balance between being the patient’s or the society’s doctor.

We propose that relevant authorities try to reduce this disparity by proposing a guideline for managing patients with borderline vitamin B12 deficiency. Ideally there would agreement upon both the upper limit of serum B12 levels where neither treatment nor complementary testing are indicated, and lower limit of B12 where treatment is indicated without complementary testing. Recommendations should also be made for managing patients with levels in-between the cut-off values, perhaps driven by the presenting symptom. The guideline should then be validated in a separate study to assess the predictive values of the different cut-offs. Public funding would be essential to the development of this guideline however, as pharmaceutical companies are unlikely to give priority to vitamin B12 since it is a naturally occurring biomolecule.

## Data Availability

The authors declare that the data supporting the findings of this study are available within the article. Data for the statement regarding when last formal education was received are available from the corresponding author on reasonable request.
